# N-States Continuous Maxwell Demon

**DOI:** 10.3390/e25020321

**Published:** 2023-02-09

**Authors:** Paul Raux, Felix Ritort

**Affiliations:** 1 Université Paris Cité, CNRS, UMR 8236-LIED, 75013 Paris, France; 2 Université Paris-Saclay, CNRS/IN2P3, IJCLab, 91405 Orsay, France; 3 Small Biosystems Lab, Condensed Matter Physics Department, University of Barcelona, 08028 Barcelona, Spain; 4 Institut de Nanociència i Nanotecnologia (IN2UB), Universitat de Barcelona, 08028 Barcelona, Spain

**Keywords:** Maxwell demon, information-to-work conversion, correlated measurements

## Abstract

Maxwell’s demon is a famous thought experiment and a paradigm of the thermodynamics of information. It is related to Szilard’s engine, a two-state information-to-work conversion device in which the demon performs single measurements and extracts work depending on the state measurement outcome. A variant of these models, the continuous Maxwell demon (CMD), was recently introduced by Ribezzi-Crivellari and Ritort where work was extracted after multiple repeated measurements every time that *τ* is in a two-state system. The CMD was able to extract unbounded amounts of work at the cost of an unbounded amount of information storage. In this work, we built a generalization of the CMD to the N-state case. We obtained generalized analytical expressions for the average work extracted and the information content. We show that the second law inequality for information-to-work conversion is fulfilled. We illustrate the results for N-states with uniform transition rates and for the *N* = 3 case.

## 1. Introduction

**Figure 1:**
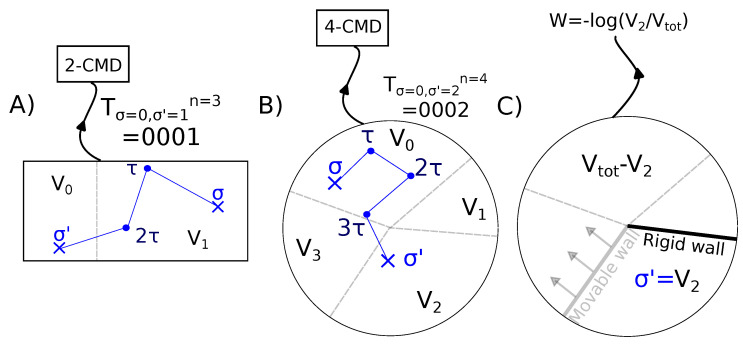
(**A**) The 2-CMD is represented as a two-compartment box in which a work extraction protocol is implemented (see text). The cycle of measurement is here $T_{0,1}^{3}=\underset{n=3}{\underset{︸}{1,1,1}},0$. The average work extracted for this cycle is $-\log\frac{V_{1}}{V}$. (**B**) 4-CMD in circular geometry. Each compartment had volume $V_{i}$. The cycle of measurement of the CMD reads: $T_{0,2}^{4}=\underset{n=4}{\underset{︸}{0,0,0,0}},2$. The initial state σ is 0 and the final state $\sigma^{\prime }$ is 2, the crossing of compartment 3 remained unnoticed for measurements made at every time τ. (**C**) In the work extraction protocol, a pair of walls limiting the volume of the last compartment, here $V_{2}$, are inserted. The wall between compartments 1 and 2 is fixed, whereas the wall between compartments 2 and 3 was movable and had no mass. To extract the work produced by the expansion of the particle confined in 2, the movable wall is connected to a pulley device. The average work extracted for this cycle is $-k_{B}T\log\frac{V_{2}}{V}$.

In 1867, James Clerk Maxwell proposed a thought experiment for the better understanding of the scope and limitations of the second law [1]. Known as the Maxwell demon paradox, it has spurred strong research activity for many years, setting the basis for the thermodynamics of information and information-to-work conversion [2,3,4,5,6,7,8,9,10]. In 1929, Leo Szilard introduced a simple physical model [11] in which a particle was free to move in a box of volume V with two compartments (denoted with 0 and 1) of volumes $V_{0}$ and $V_{1}$ and $V=V_{0}+V_{1}$ (Figure 1A). In Szilard’s engine (SZ), the “demon” was an entity able to monitor the particle’s position and store the observed compartment in a single-bit variable σ=0,1. Information-to-work conversion is as follows: once the particle’s compartment is known, a movable wall is inserted between the two compartments, and an isothermal process is implemented to extract work. A work-extracting cycle concludes when the movable wall reaches its far end, and the measurement-work extraction process restarts. The average work extracted per cycle equals the equivalent heat transferred from the isothermal reservoir to the system: $W_{2}^{Sz}=-k_{B}T\left(P_{0}\log\left(P_{0}\right)+P_{1}\log\left(P_{1}\right)\right)$, with $P_{0,1}=V_{0,1}/V$ the occupancy probabilities of each compartment. For equal compartments $P_{0}=P_{1}=1/2$, Szilard’s engine can extract maximal work determined by the Landauer bound, $W^{SZ}\leq k_{B}T\log\left(2\right)$ from the reservoir without energy consumption, meaning that heat was fully converted into work, apparently violating Kelvin’s postulate. In the 1960s and 1970s, work by Landauer [12] and Bennett [13] found a solution to the paradox. The solution to this paradox considers the information content of the measurement, the work extraction, and the resetting processes of the demon [14,15]. Most importantly, to recover the initial state at the end of the thermodynamic cycle, the demon must erase the information acquired on the system [2]. The minimal erasure cost per bit of information equals $k_{B}T\log\left(2\right)$ for equally probable outcomes. In the end, the information content stored in the demon is always larger than or equal to the extracted work, in agreement with the second law.

In a recent paper, a new variant of the Maxwell demon, the continuous Maxwell demon (CMD), was introduced [16] (see also [17] for additional results), analytically solved, and experimentally tested. In the CMD, the demon performs repeated measurements of a particle’s location in a two-compartment box every time τ. The first time that the demon measures that the particle changed compartments, a work extraction procedure is implemented. The main difference with the SZ engine is that, in the CMD, a work extraction cycle contains multiple measurements, whereas for the SZ, a single measurement is performed at every work cycle. Compared to the SZ, the CMD can extract a more significant amount of work *W* because of the larger information content of the multiple stored bits in a cycle. Interestingly, the average work per cycle in the CMD satisfies $W^{CMD}\geq k_{B}T\log\left(2\right)$ being unbounded in the limits $P_{0}\to 0$ ($P_{1}\to 1$) and $P_{0}\to 1$ ($P_{1}\to 0$). A model combining the SZ and CMD work extraction protocols version showed the role of temporal correlations in optimizing information-to-energy conversion [18]. In the CMD, the time between measurements τ is arbitrary. In particular, it can be made infinitesimal, τ→0, leading to an infinite number of measurements per cycle justifying the *continuous* adjective of the model.

Here, we generalize the CMD to the case of N-states (*N*-CMD). In a possible realization of the *N*-CMD, a particle in a box of volume *V* can occupy *N* distinct compartments of volumes $V_{i}$ (Figure 1B). The demon measures in which compartment the particle is at every time τ until a change in the compartment is detected. Then, a work extraction process is implemented by inserting one fixed wall at one side and one movable wall at the other side of the compartment that can expand under the elastic collisions exerted by the particle (Figure 1C). A pulley mechanism is attached to the movable wall to extract an average work equal to $W=-k_{B}T\log P_{i}$ with $P_{i}=V_{i}/V$. For *N* = 2, we obtain the standard CMD (Figure 1A) (2-CMD), which corresponds to transforming the Szilard box (Figure 1B) into a periodic torus (Figure 1C).

The outline of this work is as follows. In Section 2, we show how to generalize the mathematical formalism of the 2-CMD to *N* states (*N*-CMD). In Section 3, we analyze the performance of the *N*-CMD by studying the thermodynamic efficiency and power. In Section 4, we analyze several cases, and in particular case N=3, to investigate the effect of topology on information-to-work conversion (IWC). We end with some conclusions and future directions.

## 2. General Setting

Let σ (=1,…,N) denote the *N* states of a system following Markovian dynamics defined by transition rates that satisfy detailed balance, ensuring that the system relaxes to an equilibrium state. Let τ be the time between consecutive measurements. The conditional probability $p_{\tau }\left(\sigma^{\prime }|\sigma \right)$ that the outcome of the measurement is $\sigma^{\prime }$ after time τ conditioned that it starts in σ satisfies the following master equation:

(1) $$\partial_{\tau }p_{\tau }\left(\sigma^{\prime }|\sigma \right)=\sum_{\sigma^{\prime \prime }=1}^{N}K_{\sigma^{\prime }\sigma^{\prime \prime }}p_{\tau }\left(\sigma^{\prime \prime }|\sigma \right)$$

with initial condition $p_{\tau \to 0}\left(\sigma^{\prime }|\sigma \right)\to \delta_{\sigma^{{}^{\prime }},\sigma }$, where δ is the Kronecker delta function. Markov matrix $K_{\sigma^{\prime }\sigma^{\prime \prime }}$ satisfies $\sum_{\sigma^{\prime }}K_{\sigma^{\prime }\sigma^{\prime \prime }}=0;\forall \sigma^{\prime \prime }$, defining transitions rates from state $\sigma^{\prime \prime }$ to $\sigma^{\prime }$:

(2) $$K_{\sigma^{\prime }\sigma^{\prime \prime }}=\left\{\begin{array}{cc} -\left(\sum_{\sigma (\neq \sigma^{\prime \prime })}k_{\sigma \leftarrow \sigma^{\prime \prime }}\right) & \;\mathrm{if}\;\sigma^{\prime }=\sigma^{\prime \prime }\\ k_{\sigma^{\prime }\leftarrow \sigma^{\prime \prime }} & \mathrm{otherwise} \end{array}\right.$$

with $k_{\sigma^{\prime }\leftarrow \sigma^{\prime \prime }}$ the probability to jump from state $\sigma^{\prime \prime }$ to $\sigma^{\prime }$ during time dτ. Let us denote by $P_{\sigma }$ the stationary solutions of Equation (1). The detailed balance condition reads:

(3) $$\forall \sigma ,\sigma^{\prime }\;;K_{\sigma \sigma^{\prime }}P_{\sigma^{\prime }}=K_{\sigma^{\prime }\sigma }P_{\sigma }$$

The solution of Equation (1) can be written using the Perron–Frobenius theorem (see [19]) as a spectral expansion in terms of the eigenvalues and eigenvectors of *K*:

(4) $$p_{\tau }\left(\sigma^{\prime }|\sigma \right)=P_{\sigma }\sum_{\alpha }l_{\sigma^{\prime }}^{\alpha }l_{\sigma }^{\alpha }\exp\left(\lambda^{\alpha }\tau \right)$$

where $l^{\alpha }$ is the left eigenvector of *K* associated with the eigenvalue $\lambda^{\alpha }$. The sum over the α term in Equation (4) is symmetric in $\sigma ↔\sigma^{\prime }$. Therefore, the conditional probabilities also fulfil a detailed balance:

(5) $$\frac{p_{\tau }\left(\sigma |\sigma^{\prime }\right)}{p_{\tau }\left(\sigma^{\prime }|\sigma \right)}=\frac{P_{\sigma }}{P_{\sigma^{\prime }}}$$

**Remark** **1.** *Detailed balance ensures that there exists a unique stationary state $P_{\sigma }$ associated with the eigenvalue $\lambda^{0}=0$ and that the other eigenvalues are real and negative, $\lambda^{\alpha \neq 0}<0$. Equation (4) can be rewritten as follows:*

(6) $$p_{\tau }\left(\sigma^{\prime }|\sigma \right)=P_{\sigma^{\prime }}\left(1+\sum_{\alpha \neq 0}l_{\sigma^{\prime }}^{\alpha }l_{\sigma }^{\alpha }\exp\left(\lambda^{\alpha }\tau \right)\right)$$

*which gives $p_{\tau }\left(\sigma^{\prime }|\sigma \right)=P_{\sigma^{\prime }}$ for τ→∞ as expected.*

In the CMD, a work-extraction cycle is defined by a sequence of n+1 measurement outcomes $\sigma_{i}$ (1≤i≤n+1) repeatedly taken every time τ. In a cycle the first *n* outcomes are equal (σ) ending with $\sigma^{\prime }$ (≠σ). We define the trajectory for a cycle as follows:

(7) $$T_{\sigma \sigma^{\prime }}^{n}=\underset{n}{\underset{︸}{\sigma ,\ldots ,\sigma ,}}\;\sigma^{\prime }$$

The probability of a given trajectory $T_{\sigma \sigma^{\prime }}^{n}$ reads:

(8) $$P\left(T_{\sigma \sigma^{\prime }}^{n}\right)=p_{\tau }{\left(\sigma |\sigma \right)}^{n-1}p_{\tau }\left(\sigma^{\prime }|\sigma \right)$$

This is normalized as follows:

(9) $$\sum_{\sigma^{\prime }\left(\neq \sigma \right)}\sum_{n=1}^{\infty }P\left(T_{\sigma \sigma^{\prime }}^{n}\right)=1\;\;,\forall \sigma$$

### 2.1. Thermodynamic Work and Information-Content

Like in the SZ, the work extracted by the CMD in a given cycle $T_{\sigma \sigma^{\prime }}^{n}$ equals $-\log(P_{\sigma^{\prime }})$. Averaging over all the possible measurement cycles, we obtain the average extracted work:

(10) $$\begin{array}{cc} W_{N}\left(\tau \right) & =<-\log P_{\sigma^{\prime }}>=-\sum_{\sigma }\sum_{\sigma^{\prime }\left(\neq \sigma \right)}\sum_{n=1}^{\infty }P_{\sigma }P\left(T_{\sigma \sigma^{\prime }}^{n}\right)\log P_{\sigma^{\prime }}\\ \; & =-\sum_{\sigma =1}^{N}\frac{P_{\sigma }}{1-p_{\tau }\left(\sigma |\sigma \right)}\sum_{\sigma^{\prime }\neq \sigma }p_{\tau }\left(\sigma^{\prime }|\sigma \right)\log P_{\sigma^{\prime }} \end{array}$$

which is positive by definition. In the limit τ→∞ we obtain the following expression,

(11) $$W_{N}^{\infty }=-\sum_{\sigma =1}^{N}\frac{P_{\sigma }}{1-P_{\sigma }}\sum_{\sigma^{\prime }\neq \sigma }P_{\sigma^{\prime }}\log P_{\sigma^{\prime }},$$

which can be written as follows:

(12) $$W_{N}^{\infty }=(\sum_{\sigma =1}^{N}\frac{P_{\sigma }}{1-P_{\sigma }})W_{N}^{\mathrm{SZ}}+\sum_{\sigma =1}^{N}\frac{P_{\sigma }^{2}\log P_{\sigma }}{1-P_{\sigma }}$$

where $W_{N}^{SZ}$ is the classical statistical entropy of the system, which can also be interpreted as the average work extraction of the *N*-states Szilard engine, denoted as *N*-SZ:

(13) $$W_{N}^{\mathrm{SZ}}=<-\log\left(P_{\sigma }\right)>=-\sum_{\sigma }P_{\sigma }\log P_{\sigma }$$

This expression can be readily minimized in the space of $P_{\sigma }$ giving the uniform solution, $P_{\sigma }=1/N$ for which $W_{N}^{\infty }=\log N$. In contrast, $W_{N}^{\infty }\to -\log\left(1-P_{\sigma }\right)$ if $P_{\sigma }\to 1$ for a given σ (and $P_{\sigma^{\prime }}\to 0$ $\forall \sigma^{\prime }\neq \sigma$) diverging in that limit.

We define the average information content per cycle as the statistical entropy of the measurement-cycle probabilities [20]:

(14) $$\begin{array}{cc} I_{N}\left(\tau \right) & =<-\log\left(P_{\sigma }P\left(T_{\sigma \sigma^{\prime }}^{n}\right)\right)>\\ \; & =-\sum_{\sigma }\sum_{\sigma^{\prime }\left(\neq \sigma \right)}\sum_{n=1}^{\infty }P_{\sigma }P\left(T_{\sigma \sigma^{\prime }}^{n}\right)\log\left(P_{\sigma }P\left(T_{\sigma \sigma^{\prime }}^{n}\right)\right)\\ \; & =-\sum_{\sigma =1}^{N}\sum_{\sigma^{\prime }\neq \sigma }^{N}P_{\sigma }\left(p_{\tau }\left(\sigma^{\prime }|\sigma \right)\log\left(P_{\sigma }\right)+p_{\tau }\left(\sigma^{\prime }|\sigma \right)\log\left(p_{\tau }\left(\sigma^{\prime }|\sigma \right)\right)\right)\underset{\frac{1}{1-p_{\tau }\left(\sigma |\sigma \right)}}{\underset{︸}{\sum_{n=1}^{\infty }p_{\tau }{\left(\sigma |\sigma \right)}^{n-1}}}\\ \; & -\sum_{\sigma =1}^{N}\sum_{\sigma^{\prime }\neq \sigma }^{N}P_{\sigma }p_{\tau }\left(\sigma^{\prime }|\sigma \right)\log\left(p_{\tau }\left(\sigma |\sigma \right)\right)\underset{\frac{p_{\tau }\left(\sigma |\sigma \right)}{{\left(1-p_{\tau }\left(\sigma |\sigma \right)\right)}^{2}}}{\underset{︸}{\sum_{n=1}^{\infty }\left(n-1\right)p_{\tau }{\left(\sigma |\sigma \right)}^{n-1}}}\\ \; & =W_{N}^{SZ}-\sum_{\sigma }^{N}\frac{P_{\sigma }}{1-p_{\tau }\left(\sigma |\sigma \right)}\sum_{\sigma^{\prime }}p_{\tau }\left(\sigma^{\prime }|\sigma \right)\log p_{\tau }\left(\sigma^{\prime }|\sigma \right) \end{array}$$

The positivity of $I_{N}\left(\tau \right)$ follows from the fact that $p_{\tau }\left(\sigma^{\prime }|\sigma \right),P_{\sigma }\leq 1$. The second term in Equation (14) depends on τ and can be understood as the contribution of correlations between measurements to $I_{N}\left(\tau \right)$.

Lastly, using Equation (5), we can rearrange Equation (14) as follows:

(15) $$I_{N}\left(\tau \right)=W_{N}\left(\tau \right)\underset{=\Delta_{N}>0}{\underset{︸}{-\sum_{\sigma }^{N}\frac{P_{\sigma }}{1-p_{\tau }\left(\sigma |\sigma \right)}\sum_{\sigma^{\prime }}p_{\tau }\left(\sigma^{\prime }|\sigma \right)\log p_{\tau }\left(\sigma |\sigma^{\prime }\right)}}$$

where the second term $\Delta_{N}$ is positive since $p_{\tau }\left(\sigma |\sigma^{\prime }\right)\leq 1$. Equation (15) implies the second law inequality:

(16) $$I_{N}\left(\tau \right)-W_{N}\left(\tau \right)>0\;\forall \tau$$

meaning that the cost to erase the stored sequences information content is always larger than the work extracted by the demon.

### 2.2. Comparison with the Szilard Engine

Equating Expressions (14) and (15) for $I_{N}\left(\tau \right)$, we obtain a relation between $W_{N}\left(\tau \right)$ and $W^{SZ}$ that compares the average work extracted in the *N*-CMD to the *N*-SZ engine as follows:

(17) $$\begin{array}{cc} W_{N}\left(\tau \right)-W_{N}^{SZ} & =<-\log\frac{P_{\sigma^{\prime }}}{P_{\sigma }}>\\ \; & =-\sum_{\sigma }\frac{p_{\sigma }}{1-p_{\tau }\left(\sigma |\sigma \right)}\sum_{\sigma^{\prime }}p_{\tau }\left(\sigma^{\prime }|\sigma \right)\log\frac{p_{\tau }\left(\sigma^{\prime }|\sigma \right)}{p_{\tau }\left(\sigma |\sigma^{\prime }\right)}\geq 0 \end{array}$$

where the first equality follows from the difference between the first right-hand side of Equations (10) and (13). This shows that the CMD’s average work per cycle is always larger or equal to SZ. The equality holds for the uniform case $P_{\sigma }=1/N$ where $W_{N}\left(\tau \right)=W_{N}^{SZ}=\log N$.

## 3. Thermodynamic Power and Efficiency

### 3.1. Average Cycle Length

As a preliminary, we first compute the average time of a cycle of measurement. This is similar to the mean first residence time of the system, except for the fact that (unobserved) hopping events are permitted. We define it as follows:

(18) $$t_{N}^{c}\equiv \tau <n>$$

and obtain the following expression:

(19) $$t_{N}^{c}=\tau \left(1+\sum_{\sigma }\frac{P_{\sigma }}{1-p_{\tau }\left(\sigma |\sigma \right)}\right)$$

The following equalities are shown:

(20) $$\lim_{\tau \to 0^{+}}t_{N}^{c}=-\sum_{i}\frac{1}{\sum_{\alpha \neq 0}{\left(l_{i}^{\alpha }\right)}^{2}\lambda^{\alpha }}>0$$

(21) $$\lim_{\tau \to \infty }t_{N}^{c}=+\infty$$

The average cycle time is the mean first passage time [21] of the discrete time random walk defined by a cycle of measurements.

### 3.2. Thermodynamic Power

We define the thermodynamic power as the average work $W_{N}$ extracted per cycle time $t_{N}^{c}$:

(22) $$\Phi_{N}\left(\tau \right)=\frac{W_{N}}{t_{N}^{c}}$$

In the limit of uncorrelated measurements τ→∞, we obtain from Equations (11) and (19):

(23) $$\Phi_{N}^{\infty }=-\frac{1}{\tau }\frac{\sum_{\sigma =1}^{N}\frac{P_{\sigma }}{1-P_{\sigma }}\sum_{\sigma^{\prime }\neq \sigma }P_{\sigma^{\prime }}\log P_{\sigma^{\prime }}}{1+\sum_{\sigma }\frac{P_{\sigma }}{1-P_{\sigma }}}$$

For N=2, we recover the results in [16,17].

### 3.3. Information-to-Work Efficiency

In the spirit of the efficiencies defined for thermal machines, we define the information-to-work conversion (IWC) efficiency of the CMD as the ratio between $W_{N}$, taken to be the objective function, and $I_{N}$, taken to be the cost function, for the optimization of the CMD:

(24) $$\eta_{N}=\frac{W_{N}}{I_{N}}$$

Using Equation (15), we can rewrite $\eta_{N}$ as follows:

(25) $$\eta_{N}=\frac{1}{1+\frac{\Delta_{N}}{W_{N}}}$$

From Equation (16), $\eta_{N}<1$. In the limit τ→∞, we obtain:

(26) $$\lim_{\tau \to \infty }\eta_{N}=\frac{1}{1+\frac{\sum_{i}\frac{P_{i}}{1-P_{i}}\log P_{i}}{\sum_{i}\frac{P_{i}}{1-P_{i}}\sum_{j\neq i}P_{j}\log P_{j}}}$$

In limit $P_{\sigma }\to 1$ for a given state σ, one can check that the *N*-CMD reaches maximal efficiency 1.

## 4. Particular Cases

Here, we analyze some specific examples.

### 4.1. Case N = 2

We now turn to the N=2 case considered in [16] as an example of our formulae. The kinetic rate matrix in this case reads:

(27) $$K=\left(\begin{array}{cc} -k_{1\leftarrow 0} & k_{0\leftarrow 1}\\ k_{1\leftarrow 0} & -k_{0\leftarrow 1} \end{array}\right)$$

Here, we do not need to make any particular choice of rates $k_{\sigma^{\prime }\sigma }$ to ensure detailed balance since, for two states, a detailed balance unconditionally holds. Applying the procedure sketched in Section 2, we solve the master equation:

(28) $$p_{\tau }={\left(p_{\tau }\left(\sigma |\sigma^{\prime }\right)\right)}_{\sigma ,\sigma^{\prime }=0,1}\left(\begin{array}{cc} P_{0}+P_{1}\exp\left(-R\tau \right) & P_{0}\left(1-\exp\left(-R\tau \right)\right)\\ P_{1}\left(1-\exp\left(-R\tau \right)\right) & P_{1}+P_{0}\exp\left(-R\tau \right) \end{array}\right)$$

where $R=k_{1\leftarrow 0}+k_{0\leftarrow 1}$, $P_{0}=\frac{k_{0\leftarrow 1}}{R}$ and $P_{1}=\frac{k_{1\leftarrow 0}}{R}$ such that $P_{0}+P_{1}=1$. $p_{\tau }$ is normalized per column:

(29) $$p_{\tau }\left(\sigma^{\prime }|\sigma \right)+p_{\tau }\left(1-\sigma^{\prime }|\sigma \right)=1\;,\;\;\forall \sigma =0,1$$

First, let us consider $W_{2}$. Since N=2 and by normalization, there is only one term in the sum $\sum_{\sigma^{\prime }\neq \sigma }$ of Equation (10). Thus, $W_{2}$ simplifies to:

(30) $$W_{2}=-P_{0}\log\left(1-P_{0}\right)-\left(1-P_{0}\right)\log\left(P_{0}\right)$$

We recover the result obtained in [16] and coincidently show that the τ independence of this result is a particular feature of the N=2 case. Moreover, since $W_{2}$ had a simple expression, we obtained a tractable expression for the comparison with the SZ average work extracted, c.f. Equation (17):

(31) $$W_{2}-W_{2}^{SZ}=\left(1-2P_{0}\right)\log\left(\frac{1-P_{0}}{P_{0}}\right)$$

This quantity is positive and vanishes only for uniform probability, $P_{\sigma }=\frac{1}{2}$, as shown in Section 3.1. Using normalization Equation (29) again in the definition of $I_{N}$ Equation (14), we obtain $I_{2}$ as follows:

(32) $$\begin{array}{cc} I_{2} & =-P_{0}\log P_{0}-\left(1-P_{0}\right)\log\left(1-P_{0}\right)\\ \; & -P_{0}\left(\frac{p_{\tau }\left(0|0\right)}{p_{\tau }\left(1|0\right)}\log p_{\tau }\left(0|0\right)+\log p_{\tau }\left(1|0\right)\right)\\ \; & -\left(1-P_{0}\right)\left(\frac{p_{\tau }\left(1|1\right)}{p_{\tau }\left(0|1\right)}\log p_{\tau }\left(1|1\right)+\log p_{\tau }\left(0|1\right)\right) \end{array}$$

which is the result obtained in [16]. The remaining results of [16] are obtained by combining Equations (28), (30), and (32).

### 4.2. Uniform Transition Rates

In this subsection, we take the following particular case for the Markov matrix *K*:

(33) $$K_{\sigma^{\prime }\sigma }=R\times \left\{\begin{array}{cc} -(N-1) & \;\mathrm{if}\;\sigma^{\prime }=\sigma \\ 1 & \mathrm{otherwise} \end{array}\right.$$

In this case, there are only two independent conditional probabilities; we can thus rewrite the master equation as follows:

(34) $$\partial_{\tau }p_{\tau }\left(\sigma |\sigma \right)=R\left(1-Np_{\tau }\left(\sigma |\sigma \right)\right)$$

Via normalization, we obtain $p_{\tau }\left(\sigma^{\prime }|\sigma \right)$ as follows:

(35) $$p_{\tau }\left(\sigma^{\prime }|\sigma \right)=\frac{1}{N-1}\left(1-p_{\tau }\left(\sigma |\sigma \right)\right)\;\;\;;\;\;\;\sigma^{\prime }\neq \sigma$$

In the remainder of this subsection, we define the dimensionless rescaled time between two measurements as $\tilde{\tau }=R\tau$. The solution of Equation (34) reads:

(36) $$p_{\tau }\left(\sigma |\sigma \right)=\frac{1}{N}\left(1+\left(N-1\right)\exp\left(-N\tilde{\tau }\right)\right)$$

This particular case allows for us to obtain a glimpse of the dependence of the quantities introduced in Section 2 with N. The average work extracted is as follows:

(37) $$W_{N}=\log N\;\;\;\;.$$

We see that the work extracted does not depend on τ. $I_{N}$ reads:

(38) $$I_{N}=\log N-\frac{N}{N-1}\log(\frac{1}{N}\left(1-\exp\left(-N\tilde{\tau }\right)\right))$$

The first remark is that in the limit $\tilde{\tau }\to \infty$, $I_{N}^{\infty }=\frac{2N-1}{N-1}\underset{W_{N}}{\underset{︸}{\log N}}$.

One way to optimize the CMD is to maximize IWC efficiency, defined as follows:

(39) $$\eta_{N}\equiv \frac{W_{N}}{I_{N}}=\frac{\log N}{\log N-\frac{N\log\left(\frac{1-e^{-N\tilde{\tau }}}{N}\right)}{N-1}}$$

We find the asymptotic efficiency $\eta_{N}^{\infty }=\frac{N-1}{2N-1}$ for $\tilde{\tau }\to \infty$ and $\eta_{N}=\frac{1}{2}$ for N→∞. For the thermodynamic power, we obtain:

(40) $$\Phi_{N}\equiv \frac{W_{N}}{t_{N}^{c}}=\frac{\log N}{\frac{N\tilde{\tau }e^{N\tilde{\tau }}}{\left(N-1\right)\left(e^{N\tilde{\tau }}-1\right)}+\tilde{\tau }}$$

where $t_{N}^{c}$ is the average cycle time that we analyzed in Section 3.1. One can show that the maximum thermodynamic power $\Phi_{N}=\left(N-1\right)\log\left(N\right)$ is obtained in the limit $\tilde{\tau }\to 0$. This shows that the maximum IWC efficiency Equation (39) and the efficiency at maximum power Equation (40) are obtained in two different limits, a general result expected for thermodynamic machines [22].

### 4.3. Case N = 3

**Figure 2:**
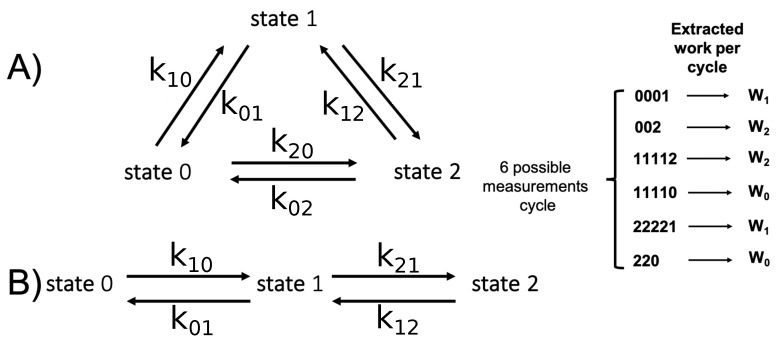
Definition of the state spaces for the 2 topologies available for the 3-CMD: (**A**) Triangular 3-CMD, (**B**) Linear 3-CMD.

The 3-CMD is the simplest case in which two different topologies of the state space are available. They are defined in Figure 2 and are denoted as triangular (Panel A) and linear (Panel B), respectively. We denote the energy of state σ(σ=0,1,2) by $\epsilon_{\sigma }$. Taking β=1, the detailed balance assumption Equation (3) then reads:

(41) $$\frac{K_{\sigma \sigma^{\prime }}}{K_{\sigma^{\prime }\sigma }}=\exp\left(-\left(\epsilon_{\sigma }-\epsilon_{\sigma^{\prime }}\right)\right)\;\;\;,\;\;\forall \sigma \neq \sigma^{\prime }$$

Here we take $\epsilon_{0}=0$. This implies that the energies of states 1,2 read:

(42) $$\epsilon_{1}=\log\left(\frac{P_{0}}{P_{1}}\right)$$

(43) $$\epsilon_{2}=\log\left(\frac{P_{0}}{P_{2}}\right)$$

In the linear case, taking as a particular case $k_{01}=1$ and $k_{21}=1$, we obtain the following Markov matrix:

(44) $$K_{3}^{lin}=\left(\begin{array}{ccc} -1 & \exp(\epsilon_{1}) & 0\\ 1 & -(\exp\left(\epsilon_{1}\right)+\exp\left(\epsilon_{1}-\epsilon_{2}\right)) & 1\\ 0 & \exp(\epsilon_{1}-\epsilon_{2}) & -1 \end{array}\right)=\left(\begin{array}{ccc} -1 & \frac{P_{0}}{P_{1}} & 0\\ 1 & -\frac{P_{0}}{P_{1}}\left(1+\frac{P_{2}}{P_{0}}\right) & 1\\ 0 & \frac{P_{2}}{P_{1}} & -1 \end{array}\right)$$

where we used Equations (42) and (43) to give an expression of $K_{3}^{lin}$ depending only on $P_{0},P_{1},P_{2}$. In the triangular case, taking $k_{01}=1$ and $k_{21}=1$ and $k_{02}=1$ as a particular case, we obtain similarly the following Markov matrix:

(45) $$K_{3}^{tri}=\left(\begin{array}{ccc} -2 & \exp(\epsilon_{1}) & \exp(\epsilon_{2})\\ 1 & -(\exp\left(\epsilon_{1}\right)+\exp\left(\epsilon_{1}-\epsilon_{2}\right)) & 1\\ 1 & \exp(\epsilon_{1}-\epsilon_{2}) & -(1+\exp\left(\epsilon_{2}\right)) \end{array}\right)=\left(\begin{array}{ccc} -2 & \frac{P_{0}}{P_{1}} & \frac{P_{0}}{P_{2}}\\ 1 & -\frac{P_{0}}{P_{1}}\left(1+\frac{P_{2}}{P_{0}}\right) & 1\\ 1 & \frac{P_{2}}{P_{1}} & -(1+\frac{P_{0}}{P_{2}}) \end{array}\right)$$

The solution of Equation (1) with Markov matrix (44) in the linear case and (45) in the triangular case, can be written using the Perron-Frobenius theorem [19] as the following spectral expansion:

(46) $$P_{\sigma }\left(\tau \right)=\Psi_{0}+c_{1}^{\sigma }\Psi_{1}\exp\left(\lambda_{1}\tau \right)+c_{2}^{\sigma }\Psi_{2}\exp\left(\lambda_{2}\tau \right)$$

where $\lambda_{1},\lambda_{2}<0$ and $c_{1}^{\sigma },c_{2}^{\sigma }$ are the coefficients determined in the limit τ→0, which depend on the conditional state σ. These coefficients are gathered in Table 1 for both models.

$\Psi_{0},\Psi_{1},\Psi_{2}$ are the eigenvectors of both $K_{3}^{lin},K_{3}^{tri}$:

- $\Psi_{0}$ is the eigenvector associated to the eigenvalue 0 and it corresponds to the stationary probability. Since the detailed balance condition Equation (3) holds, the stationary probability is the Boltzmann distribution. Thus,
  (47) $$\Psi_{0}=\left(\begin{array}{c} P_{0}\\ P_{1}\\ P_{2} \end{array}\right)=\frac{1}{Z}\left(\begin{array}{c} 1\\ \exp(-\epsilon_{1})\\ \exp(-\epsilon_{2}) \end{array}\right)$$
  where $Z=1+\exp\left(-\epsilon_{1}\right)+\exp\left(-\epsilon_{2}\right)$
- $\Psi_{1}$ is the eigenvector associated to the second eigenvalue, which reads $\lambda_{1}^{lin}=-1$ in the linear case, and $\lambda_{1}^{tri}=-\left(1+\frac{1-P_{1}}{P_{2}}\right)$ in the triangular case. $\Psi_{1}$ reads:
  (48) $$\Psi_{1}=\left(\begin{array}{c} -1\\ 0\\ 1 \end{array}\right)$$
- $\Psi_{2}$ is the eigenvector associated in both models to the eigenvalue $\lambda_{2}=-\frac{1}{P_{1}}$. It reads:
  (49) $$\Psi_{2}=\left(\begin{array}{c} \frac{P_{0}}{P_{2}}\\ -\frac{1-P_{1}}{P_{2}}\\ 1 \end{array}\right)$$

#### Uncorrelated Measurements on the 3-CMD

**Figure 3:**
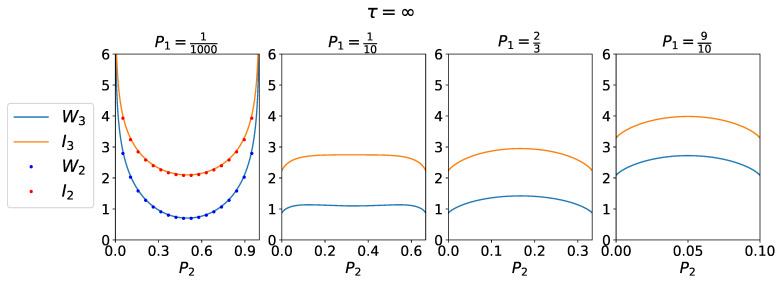
$W_{2},I_{2},W_{3},I_{3}$ as a function of $P_{2}$ for $P_{1}$ fixed in each panel. Large work extraction is obtained in the limit of rare events $P_{1}\to 0$ and $P_{2}\to 0,1$.

We now turn to the limit τ→∞. In this limit of uncorrelated measurements, the time between consecutive measurements τ is larger than the relaxation time of the system, the inverse of the lowest eigenvalue, ∼$-1/\lambda_{1}$. In this limit, $P_{\sigma }\left(\tau \right)$ reduces to Boltzmann distribution Equation (47) and $p_{\tau }\left(\sigma^{\prime }|\sigma \right)=P_{\sigma^{\prime \prime }}$. Therefore, the two models (linear and triangular) are indistinguishable. Results for work and information are shown in Figure 3.

First, it is clear that the second law Inequality (16) was satisfied. In the limit $P_{1}\to 0$, we recovered the 2-CMD. Our generalized expressions for work and information content reproduced well the trend observed in Figure 1c of [16]. In the limit of rare events, where $P_{1}\to 0$ and $P_{2}\to 0,1$, we recovered the infinite average work extraction described for the 2-CMD. Large work extraction was only obtained in the 2-CMD limit.

**Figure 4:**
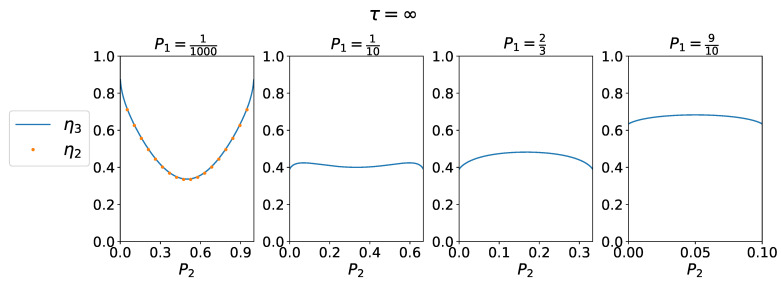
$W_{2},I_{2},W_{3},I_{3}$ as a function of $P_{2}$ for $P_{1}$ fixed in each panel.

Efficiency $\eta_{3}$ is shown in Figure 4. For $P_{1}\to 0$ and $P_{2}\to 0$ or $P_{2}\to 1$, we recovered the limit of rare events and maximal efficiency $\eta_{3}\to 1$. In the 3-CMD, we have $\eta_{3}\in \left[2/5,1\right]$.

### 4.4. Correlated Measurements in the 3-CMD

**Figure 5:**
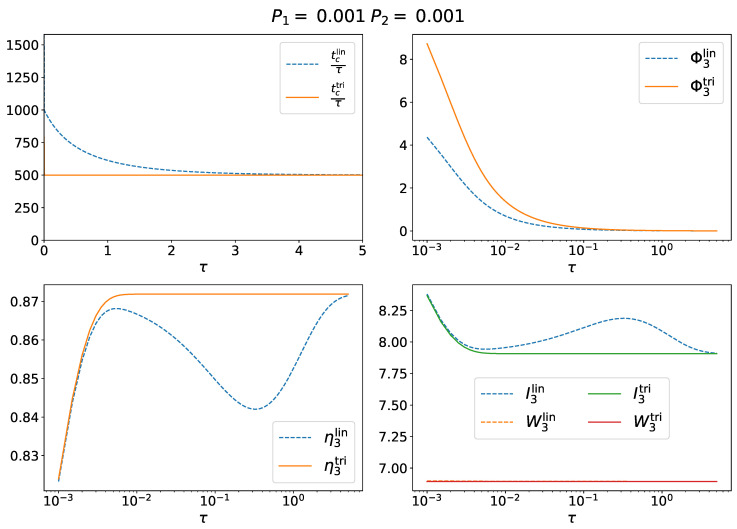
The 3-CMD for correlated measurements for $P_{1}=P_{2}=0.001$. (upper left) Average cycle length $t_{3}^{c}/\tau$ in both models, Equation (19); (upper right) thermodynamic power; (lower left) efficiency; (lower right) average information content and work extraction in $k_{B}T$ units (orange and red lines collapse on top of each other).

Correlated measurements are those where τ is lower than or comparable to the equilibrium relaxation time. Equation (46) shows that the dynamics of the linear and triangular topologies for the 3-CMD are very similar. Indeed, in the limit of uncorrelated measurements, the two dynamics reduce to the same Boltzmann distribution. They also collapse in the limit $P_{1}\to 1$ (with $P_{0},P_{2}\to 0$), indeed in this case $\lambda_{1}^{lin}=\lambda_{1}^{tri}$. In between, the topology of the network is relevant. For correlated measurements, we obtained the results shown in Figure 5.

First, the average cycle time (upper-left panel in Figure 5) in the linear case was generally larger than that in the triangular case. The direct consequence, since the average work extraction was comparable in both cases, was that the thermodynamic power (upper-right panel in Figure 5) extracted by the linear 3-CMD was lower than the thermodynamic power extracted by the triangular 3-CMD. Moreover, the thermodynamic power decreased logarithmically to 0 when τ increases. Thus, 3-CMD had optimal power production in limit τ→0, i.e., in the limit of continuous measurements. The efficiency of the 3-CMD as a function of τ is plotted in the lower-left panel of Figure 5. The linear 3-CMD was generally less efficient than the triangular 3-CMD. The reason for this is in the lower-right panel of Figure 5, where $W_{3}$ and $I_{3}$ are plotted against τ for both models. For a comparable work extraction, the linear 3-CMD needs to store more information. Again, in the limit of uncorrelated measurements, the two models converge to the same result.

## 5. Concluding Remarks

In this work, we generalized the 2-CMD of [16,17] to N-states. We obtained generalized expressions of the average extracted work, the average information content stored in the demon’s memory, and of thermodynamical quantities such as the thermodynamic power and the information-to-work efficiency of the *N*-CMD. We proved that the second law inequality holds for the *N*-CMD, thus giving bounds on the efficiency of the engine. Comparing the *N*-CMD to the *N*-SZ engine, we also showed that the *N*-CMD could extract more work on average than the *N*-SZ engine. The most efficient setting of the *N*-CMD was in the limit of rare events already described in [16]. In the *N*-CMD case, this limit was obtained by first taking the 2-CMD limit. Thus, no configuration is more efficient in the *N*-CMD than the 2-CMD limit.

In future work on the *N*-CMD, it would be interesting to implement a graph theoretic procedure to obtain, for instance, a more precise explanation of the difference between the linear and triangular cases (connected graph versus fully connected graph). It would also be interesting to determine the distributions of the quantities computed here [23] and thus optimize the fluctuations of the *N*-CMD.

## Figures and Tables

**Table 1:** Coefficients of the spectral expansion Equation (46).

	$c_{1}^{\sigma }$	$c_{2}^{\sigma }$
σ=0	$-(P_{2}+\frac{P_{2}^{2}+P_{2}\left(P_{0}+P_{1}\right)}{P_{0}+P_{2}})$	$\frac{P_{1}P_{2}}{P_{0}+P_{2}}$
σ=1	0	$\frac{P_{2}\left(P_{1}-1\right)}{P_{0}+P_{2}}$
σ=2	$\frac{P_{0}}{P_{0}+P_{2}}$	$\frac{P_{1}P_{2}}{P_{0}+P_{2}}$

## Data Availability

Data is available upon contacting the authors.
